# Prize Essay

**Published:** 1853-04

**Authors:** 


### PRIZE ESSAY.

The Mississippi Valley Association offer for the best essay on Dental Surgery $50. We hope there may be several competitors for this prize. We refer, for particulars, to the resolution of the society, found in the proceedings published in the present number of the Register.

Dr. S. D. M. is informed that his article on "Springing of Plates," was mislaid and not found until too late to go in the present number. It will appear in the next.

